# Practical Implications of KRAS Mutation Status and Sidedness of Primary Tumour in Patients with Colorectal Cancer and Synchronous Liver Metastases: A Subset Analysis of the CoSMIC Study

**DOI:** 10.3390/cancers14194833

**Published:** 2022-10-03

**Authors:** Anthony K. C. Chan, Ajith K. Siriwardena

**Affiliations:** Regional Hepato-Pancreato-Biliary Unit, Manchester Royal Infirmary, Manchester M13 9WL, UK

**Keywords:** colorectal cancer, liver metastases, KRAS

## Abstract

**Simple Summary:**

Patients presenting with liver metastases at the time of diagnosis of their colorectal cancer (termed ‘synchronous disease’) have a worse outcome than those whose disease is limited to the primary bowel tumour. There is evidence to show that patients with tumours on the right side of the colon have worse survival. Furthermore, mutations in the KRAS gene have also been shown to adversely affect outcome. This study explores the association between the side of the primary colorectal tumour in patients with synchronous disease and mutations in the KRAS gene on survival. We analyse a specific cohort of patients from the previously published CoSMIC study who presented with colorectal cancer and synchronous liver metastases and who had their colorectal tumour analysed for KRAS mutations. We find that for this cohort, neither mutations in the KRAS gene nor the side of the colon tumour influenced survival.

**Abstract:**

Patients with colorectal cancer presenting with synchronous liver metastases have less favourable outcomes than those with primary-only disease. There is evidence of different genetic mutational signatures according to the sidedness of the primary tumour. KRAS mutations are key driver mutations in colorectal cancer progression. This post hoc analysis of the previously reported CoSMIC inception cohort explores the association between primary tumour sidedness and KRAS mutational status on the outcome of patients with colorectal cancer and synchronous liver metastases. Patients diagnosed with synchronous disease were recruited between April 2014 and March 2017 and, after exclusions, 83 patients undergoing colorectal primary KRAS mutation testing constituted the final study population. Data were collected prospectively on demographic profiles, treatment, and outcomes. Twenty-one patients (25%) had right-sided tumours and 62 (75%) had left-sided tumours, with 46 (55%) and 37 (45%) exhibiting wildtype and mutated KRAS, respectively. There was no difference in distribution of liver metastases by KRAS status (unilobar vs. bi-lobar; *p* = 0.58; Fisher’s Exact test) and no difference in 5-year survival according to KRAS mutation status (Log-rank test, *p* = 0.82) or tumour sidedness (*p* = 0.16). In summary, in this cohort of patients with colorectal cancer and synchronous liver metastases, neither KRAS mutation status nor tumour sidedness influenced survival.

## 1. Introduction

Up to a fifth of patients with colorectal cancer present with synchronous liver metastases [1]. In the subset with both a resectable primary tumour and operable liver metastases, there are two principal management decisions to be made at the time of presentation [2,3]. First, should systemic chemotherapy precede surgery? Second, what is the optimum sequence of surgery—either hepatic resection and colorectal resection at a single operative procedure or as a staged approach [4,5]? Staged approaches can include the classical bowel-first surgical approach or the more recent liver-first strategy [4].

Inherited susceptibility is responsible for about one-third of cases of colorectal cancer and chromosomal instability (CIN), mismatch repair/microsatellite instability/(MMR/MSI) and cytosine and guanine (CpG) island methylation phenotype (CIMP) are the pathways through which most colorectal cancers develop [6].

In this regard, left-sided and right-sided colon cancers have different clinical and biologic characteristics [7]. Right-sided colon cancers are more likely to have genome-wide hypermethylation via the CpG island methylator phenotype, hypermutated state via microsatellite instability and carry mutations of the Kirsten rat sarcoma virus gene (KRAS) and the v-Raf murine sarcoma viral oncogene homolog gene (BRAF) [7]. These latter mutations are part of the RAS/mitogen activated Protein (MAP) kinase pathway and result in resistance to epidermal growth factor receptor inhibitor (EGRFi) therapy [8].

The North American Comprehensive Cancer Network (NCCN) recommends determination of MMR and MSI status together with assessment of RAS, RAF and human epidermal growth factor 2 (HER 2) amplification status as part of the work-up of patients with primary colorectal cancer [3]. Mutation status has become established in clinical practice in relation to selection of biologic therapy and in assessment of prognosis.

In contrast, there is limited assessment of the role of mutation status in determining treatment in patients with colorectal cancer and synchronous liver metastases. Management in the setting of patients with synchronous disease is influenced by patient fitness and mode of presentation. Patients with an urgent presentation require intervention directed at the primary tumour. Morphological distribution of disease and patient fitness/preference govern the decisions for surgery as the first intervention or systemic chemotherapy. Thus, the aim of this study was to explore the association of primary tumour sidedness and KRAS mutational status in patients presenting with colorectal cancer and synchronous liver-limited metastases.

## 2. Materials and Methods

### 2.1. Design

This study is a post hoc analysis of the CoSMIC inception cohort [1]. CoSMIC was a prospective study of patients with colorectal cancer and synchronous liver metastases recruited at the point of referral to a specialist liver surgery service (the study inception point). Between 1 April 2014 and 31 March 2017, of 599 new referrals of patients with colorectal hepatic metastases to this service, 238 (40%) were patients with colorectal cancer and synchronous liver metastases. After exclusions, 125 patients provided data, and of these, 83 underwent KRAS mutation testing and constitute the final study population [1].

### 2.2. Setting

The study took place in the regional hepatobiliary service of the Manchester Royal Infirmary, Manchester, UK. This is a tertiary hepatobiliary centre serving a population of 3.2 million.

### 2.3. Patients

Inclusion and exclusion criteria for the main CoSMIC study have been reported in detail elsewhere [9]. In brief, patients were over 18 years of age, able to give informed consent and presented with colorectal cancer and synchronous liver metastases. There was biopsy proof of malignancy from the primary tumour and no pre-resection histology from liver lesions.

### 2.4. Definitions Used in This Study

A colonic primary tumour involving any part of the caecum, ascending colon or the transverse colon up to the splenic flexure was classed as ‘right-sided’ for the purposes of this study [10]. Left-sided tumours were those involving the descending colon, sigmoid colon, the rectosigmoid junction or rectum. For the purposes of this study, rectal cancer was included with left-sided tumours.

### 2.5. Data Collection

Data were collected prospectively on demographic profiles including age, gender, mode of presentation (elective or emergency), location of primary disease, nodal status of primary tumour and status of liver metastases (solitary or multiple; unilobar or bi-lobar). In addition, information was collected on treatment including surgical sequence and use of chemotherapy with specific reference to the use of EGFR inhibitor therapy. Data were also collected on peri-operative morbidity, in-patient, all-cause mortality up to 90 days after surgery. Survival data were collected for at least 5 years after surgery. Patients were followed-up in out-patient clinic and time of first recurrence was accepted as first computed tomographic (CT) or magnetic resonance scan (MR) evidence of new lesions or disease progression. Disease progression was defined according to RECIST 1.1 [11].

### 2.6. KRAS, NRAS and BRAF Mutation Testing

All patients who presented de novo to the regional hepatobiliary service at this hospital underwent mutation testing of their primary colorectal tumour as part of their diagnostic workup. DNA was extracted from the primary tumour following surgical resection, and mutation testing was undertaken using Next Generation Sequencing at a regional genetics service (Manchester Centre for Genomic Medicine, Manchester, UK).

### 2.7. Data Analysis

The primary aim was to compare the survival of patients according to KRAS mutation status and colonic primary sidedness using Kaplan–Meier survival functions. All analyses were performed in RStudio: Integrated Development for R (PRB, Boston, MA, USA).

### 2.8. Ethics

The CoSMIC study was approved by the NHS Research Ethics Committee North West (Greater Manchester Central, Manchester, UK) (REC reference 14/NW/1397).

## 3. Results

### 3.1. Demographic Profiles

Twenty-one patients (25%) had right-sided tumours, and sixty-two (75%) had left-sided tumours (Table 1). Of the patients with left-sided tumours, there were 21 patients with rectal tumours. In terms of synchronous hepatic metastatic disease, 55 (66%) had multiple liver metastases, with 42 (51%) being bi-lobar.

### 3.2. Overview of Mutation Testing Status

KRAS mutation status was available for 83 patients, of whom 60 (48%) also underwent NRAS mutation testing. BRAF mutation testing was undertaken in 26 patients (31%) concurrently with KRAS. There were 25 patients (30%) that underwent KRAS, NRAS and BRAF mutation testing.

### 3.3. KRAS Mutation Status

Of 83 patients who underwent KRAS mutation testing, 46 (55%) had wild-type and 37 (45%) had mutated KRAS (Table 1). Fourteen of 21 patients with right-sided tumours had mutant KRAS status. There was no difference in liver disease distribution by KRAS mutation status (unilobar vs. bi-lobar; *p* = 0.58; Fisher’s Exact Test).

### 3.4. NRAS and BRAF Mutation Status

All sixty (100%) patients who underwent mutation testing for NRAS had wild type. All 26 (100%) patients who underwent mutation testing for BRAF had wild type.

### 3.5. Treatment

There was no difference between synchronous and staged surgery by KRAS mutational status (*p* = 0.89; Fisher’s exact test). Synchronous surgery was undertaken in 8 (10%) and bowel-first, staged surgery in 55 (66%) (Table 2).

Eighty (96%) received systemic chemotherapy with 47 (57% of cohort) receiving this in neoadjuvant mode. Seventeen (20%) had EGFRi therapy including one patient who was KRAS mutant status.

### 3.6. Outcomes

**Figure 1:**
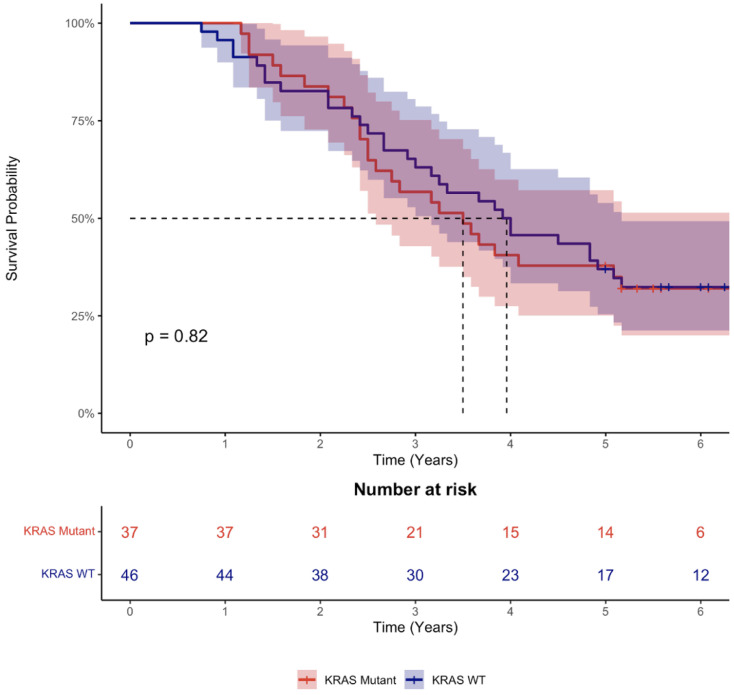
Kaplan–Meier graph of survival by KRAS mutation status. Data are presented as median with 95% confidence interval.

There was no difference in survival between patients according to KRAS mutation status (Log-rank test, *p* = 0.82) (Figure 1).

**Figure 2:**
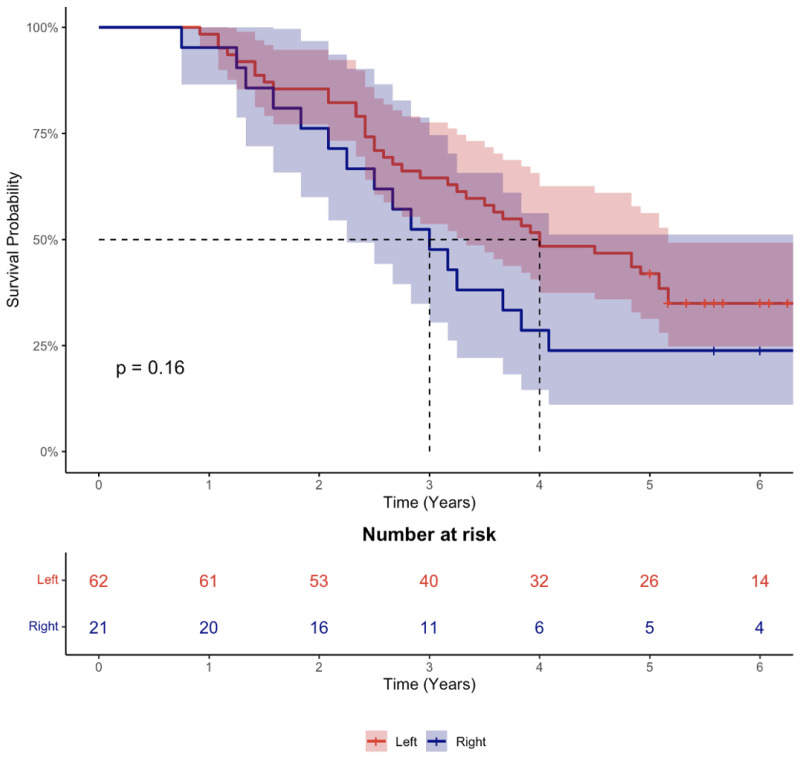
Kaplan–Meier plot of survival by tumour sidedness. Data are presented as median with 95% confidence interval.

Patients with right-sided tumours exhibited a similar survival to those with left-sided tumours (Log-rank test; *p* = 0.16) (Figure 2).

**Figure 3:**
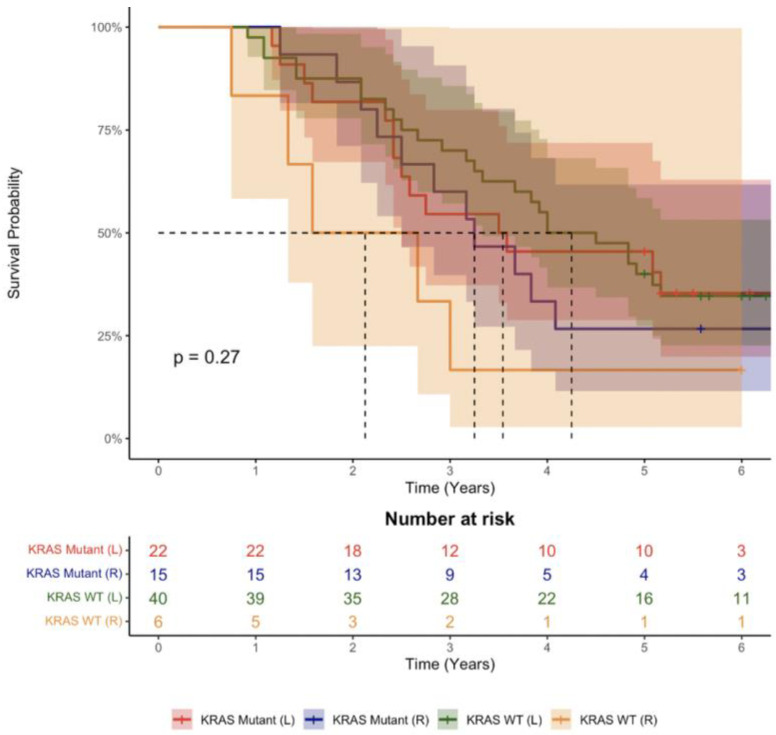
Kaplan–Meier of combined KRAS mutation status and sidedness. Data are presented as median with 95% confidence interval.

After stratifying patients into four groups according to primary tumour sidedness (right vs. left) and KRAS mutation status (wild type vs. mutant), there was no difference in survival (Log-rank test *p* = 0.27) (Figure 3).

## 4. Discussion

This study has explored the role of KRAS mutation status and tumour sidedness on outcome in patients with colorectal cancer and synchronous liver metastases.

When interpreting these findings, it is first and foremost important to acknowledge limitations in the dataset. The study is small and not undertaken to an a priori power calculation based on known mutation frequency and thus the risk of type II error is high. Recruitment for mutational analysis was not undertaken as a consecutive series and thus there is a risk of both ascertainment and sampling bias [12]. The mutational analysis was restricted to that undertaken for clinical purposes and neither genome-wide data nor deep sequencing data for KRAS were available [13].

Having acknowledged these limitations, the data provide new, prospectively collected information on the interactions between KRAS mutation, sidedness and outcome in patients with colorectal cancer and synchronous liver metastases.

To set these findings in context, the mutational frequencies should be compared to known data. First, the mutation frequency for KRAS of 45% is similar to that expected for colorectal cancer [14]. KRAS mutation status can be incorporated into the pre-operative information gathered to calculate a clinical risk score which influences decision making between upfront surgery and systemic chemotherapy. The conventional clinical risk score is based on the time interval between primary tumour and liver metastases (and thus for patients with synchronous disease, this is always an additional point), number and size of liver metastases, nodal status of primary tumour and carcino-embryonic antigen (CEA) status [15]. Brudvik and colleagues demonstrated that the incorporation of KRAS mutation status into this score by replacing disease-free interval, number of metastases, and CEA level with RAS mutation status produced a “modified clinical-risk score” that outperformed the conventional score [16]. This score has yet to be evaluated in patients with synchronous disease, but as disease-free interval is no longer a component of the modified risk score, there is a relative indication in favour of establishing KRAS mutation status from the primary tumour in situations where this is feasible. This would allow for calculation of the modified risk score in patients with synchronous disease.

In practice, for those patients with colorectal cancer and synchronous liver metastases who present as urgencies with obstruction, perforation or bleeding, mutation status testing of the primary is not an option. However, the data presented here support the findings of the Brudvik study in suggesting that where possible, the KRAS mutation status of the primary should be established. For those with mutated KRAS, together with radiological evidence of a node-positive primary tumour and liver metastases larger than 5 cm in diameter, the evidence of adverse prognosis is sufficient to suggest that systemic chemotherapy should be used as the first step rather than surgery. Thus although 35 (95%) of patients with mutated KRAS in this study received systemic chemotherapy, this was delivered as neoadjuvant therapy in only 15 (41%). Mutation status was gathered on resected colonic tumours in this study. Thus, setting the findings in the context of the known literature argues in favour of establishment of KRAS mutation status on pre-resection biopsies. This would allow incorporation of mutation status into practical treatment decision making.

Right-sided colon cancer is also associated with a worse prognosis than a left-sided primary cancer [17]. This presents a challenge to practical clinical management as right-sided colon resection more usually avoids the need for a stoma, may be a less extensive operation, avoids pelvic dissection and thus from a purely morphological perspective may be more readily combined with a liver resection [4]. However, if the worse prognosis of right-sided cancer is combined with KRAS mutation status obtained from biopsy material a practical evidence-based recommendation would be to consider systemic treatment as the first intervention in patients with right-sided cancer, mutated KRAS and synchronous liver metastases.

What of the other genes assessed in this study? Although none of the sixty patients tested carried mutations of NRAS this is compatible with the known mutation frequency in colorectal cancer of about 3% [14].

An important limitation of this study is the lack of information on other genetic and epigenetic mutations known to influence outcome. Specifically, it would be invaluable to have information on tumours demonstrating deficient mismatch repair (dMMR) [18]. This information is particularly pertinent in view of the very recent findings of the NICHE 2 study [19]. This potentially practice-changing study confirms the previously reported pathologic responses to short-term neoadjuvant nivolumab plus ipilimumab in a large cohort of dMMR colorectal cancer patients with the first survival data suggesting a strong survival benefit and thus a very real likelihood for neoadjuvant immunotherapy to become standard of care. This recent development is important and suggests that mismatch repair status should be established on pre-resection biopsies.

In the specific context of synchronous disease, the genetic heterogeneity of liver metastases compared to primary tumour and regional heterogeneity within metastases brings further complexity to the analysis of the influence of KRAS mutation status and sidedness [20,21]. Mutational information on metastases will not be available prior to selection of treatment in patients undergoing upfront surgery, and typically, will not be available for metastases until after hepatectomy. This is important when the differential expression of consensus molecular subtypes between primary and liver metastases is considered [22].

In current practice, management algorithms for patients with colorectal cancer and synchronous liver metastases are governed partly by the symptom profile at presentation (urgent/elective) but also by disease morphology on cross-sectional imaging [23,24].

However, there is emerging evidence that both KRAS mutation status and primary tumour location are important in determining outcome in patients with colorectal cancer and synchronous liver metastases [25]. In a pathfinding study, Chatila and colleagues analysed genomic and transcriptomic profiles of 738 untreated rectal cancers. No somatic alterations had significant associations with response to neoadjuvant therapy in a treatment-agnostic manner, but KRAS mutations were associated with faster relapse [25]. This study illustrates the need for continued acquisition of data on the role of mutation status and sidedness in treatment planning for patients with colorectal cancer.

## 5. Conclusions

In summary, this study has examined the role of KRAS mutation status and tumour sidedness on outcome in patients with colorectal cancer and synchronous liver metastases. In these data, KRAS mutation status and primary tumour sidedness did not influence outcome. The known adverse prognosis of patients with KRAS mutations and the possibility of integrating this evidence into a modified clinical risk score together with recent evidence on mismatch repair and the amenability to immunotherapy would suggest that KRAS mutation status and dMMR status be established on pre-resection biopsies of the primary tumour.

## Figures and Tables

**Table 1:** Demographic Profile.

	Total		Right		Left		Total
KRAS Status *	WT	Mutant	WT	Mutant	WT	Mutant	
*n* (%)	46 (55%)	37 (45%)	6 (7%)	15 (18%)	40 (48%)	22 (27%)	83
Median age (range), years	61 (31–86)	64 (39–77)	62 (53–86)	70 (39–77)	61 (31–81)	60 (46–75)	62 (31–86)
Sex Ratio, Male:Female	2.5	1.5	2.0	0.9	2.6	2.1	2.0
Emergency Presentation, %	11%	19%	0%	40%	13%	5%	14%
Primary Site							
*Caecum*	2	9	2	9	0	0	11
*Ascending*	1	2	1	2	0	0	3
*Transverse*	3	3	3	3	0	0	6
*Splenic*	0	1	0	1	0	0	1
*Descending*	2	1	0	0	2	1	3
*Sigmoid*	19	12	0	0	19	12	31
*Rectosigmoid*	5	2	0	0	5	2	7
*Rectum*	14	7	0	0	14	7	21
Primary Nodal Status **							
*N0*	4	5	0	3	4	2	9
*N1*	7	9	0	3	7	6	16
*N2*	14	10	2	5	12	5	24
*NX*	21	13	4	4	17	9	34
Liver Disease							
*Solitary*	18	10	5	5	13	5	28
*Multiple*	28	27	1	10	27	17	55
Liver Disease							
*Uni-lobar*	24	17	4	6	20	11	41
*Bi-lobar*	22	20	2	9	20	11	42

* WT = Wild Type; ** NX = Nodal Status indeterminate.

**Table 2:** Treatment Profile.

	Total		Right		Left		Total
KRAS Status *	WT	Mutant	WT	Mutant	WT	Mutant	
*n* (%)	46 (55%)	37 (45%)	6 (7%)	15 (18%)	40 (48%)	22 (27%)	83
Surgical Sequence (%)							
*Synchronous*	4 (9%)	4 (11%)	2 (33%)	2 (13%)	2 (5%)	2 (9%)	8 (10%)
*Bowel First*	29 (63%)	26 (70%)	2 (33%)	12 (80%)	27 (68%)	14 (64%)	55 (66%)
*Liver First*	8 (17%)	6 (16%)	0 (0%)	0 (0%)	8 (20%)	6 (27%)	14 (17%)
*No Surgery*	5 (11%)	1 (3%)	2 (33%)	1 (7%)	3 (8%)	0 (0%)	6 (7%)
Chemotherapy (%)	45 (70%)	35 (95%)	6 (100%)	14 (93%)	39 (98%)	21 (95%)	80 (96%)
*Neoadjuvant*	32 (70%)	15 (41%)	3 (50%)	5 (33%)	29 (73%)	10 (45%)	47 (57%)
Staged **	11 (30%)	20 (63%)	0 (0%)	9 (75%)	11 (31%)	11 (55%)	31 (45%)
*Adjuvant*	23 (50%)	18 (49%)	3 (50%)	7 (47%)	20 (50%)	11 (50%)	41 (49%)
EGFR Inhibitor (%)	16 (35%)	1 (3%)	2 (33%)	0 (0%)	14 (35%)	1 (5%)	17 (20%)
VEGF Inhibitor (%)	5 (11%)	1 (3%)	0 (0%)	0 (0%)	5 (13%)	1 (5%)	6 (7%)

* WT = Wild Type; ** % of staged surgery (bowel-first and liver-first) patients only.

## Data Availability

The data presented in this study are available on request from the corresponding author. The data are not publicly available due to patient confidentiality inherent to a small dataset.
